# Chemical Composition and Antimicrobial Activities of Essential Oils from *Nepeta cataria* L. against Common Causes of Food-Borne Infections

**DOI:** 10.5402/2012/591953

**Published:** 2012-06-17

**Authors:** Kamiar Zomorodian, Mohammad Jamal Saharkhiz, Samaneh Shariati, Keyvan Pakshir, Mohammad Javad Rahimi, Reza Khashei

**Affiliations:** ^1^Center of Basic Research in Infectious Disease, Shiraz University of Medical Sciences, Shiraz 71348-45794, Iran; ^2^Department of Horticultural Sciences, Faculty of Agriculture, Shiraz University, Shiraz 71441-65186, Iran; ^3^Department of Parasitology and Medical Mycology, School of Medicine, Shiraz University of Medical Sciences, Shiraz 71348-45794, Iran; ^4^Department of Medical Bacteriology and Virology, School of Medicine, Shiraz University of Medical Sciences, Shiraz 71348-45794, Iran

## Abstract

*Nepeta cataria* L. is traditionally consumed as a food additive. The effects of three different harvest stages of *N. cataria* essential oils (EOs) against most common causes of food-borne infections were evaluated by broth microdilution method as recommended by the Clinical and Laboratory Standards Institute (CLSI). The chemical composition of the EOs from *N. cataria* has been analyzed by gas chromatography/mass spectrometry (GC/MS). The analysis of the EOs indicated that 4a-**α**,7-**α**,7a-**β**-nepetalactone (55–58%) and 4a-**α**,7-**β**,7a-**α**-nepetalactone (30–31.2%) were the major compounds of the EOs at all developmental stages. The results showed that the tested EOs exhibited antimicrobial activities against the food-borne pathogens at concentrations of 0.125–2 **μ**L/mL. Based on these results, the EO of *N. cataria* can possibly be used in food products as a natural preservative agent.

## 1. Introduction

For thousands of years, aromatic plants have been used for flavoring and medicinal properties [1, 2]. These plants represent a renewable source of flavoring substances and are commonly used in food, cosmetics, and pharmaceutical products [3, 4]. It has been shown that many of these plants and their aromatic products have potential antimicrobial activities [5–8]. In tropical climates where these food-borne infections are common, they are traditionally used in food stuff for their preservative properties [3, 4]. In the recent decades, there is a great tendency towards using natural products and phytochemicals in medicine and food industries to overcome antibiotic resistance and increase the shelf life of products [8].

The family *Nepeta* (Lamiaceae) with the common Persian name “puneh” includes a large number of volatile oil plants that are wildly distributed in Europe, Asia, North America, and the mountains of tropical Africa [9]. About 67 species of this family are grown endemically in Iran. *Nepeta cataria* (Catnip), a tropical aromatic plant belonging to this family, is native to Asia and Southeast Europe. Its leaves resemblemintin appearance and the flowers are white and finely spotted with purple with a strong odor [10]. In Iran and some other countries, fresh or dried leaves and flowers of *N. cataria* are used in making sauces, soups, and cheese [11].

In folk medicine, this plant has been used for antispasmodic, carminative, stimulant, and tonic properties [11–14]. Moreover, traditionally, the tea made of its leaves is known as sedative and soporific and also is used to relive gastrointestinal and respiratory disorders such as colic, diarrhea, cough, asthma, and bronchitis [11, 12, 14]. It has been shown that many medical properties of *Nepeta* species are the characteristic of its essential oil (EO) and flavonoids. The EOs of *N. cataria* are rich in nepetalactones [15–20] and have been reported to have antibacterial [15, 17, 19], antifungal [15, 19], insecticidal [21, 22] and antioxidant activities [15]. It has also been shown that the extract of *N. cateria* has an inhibitory activity on growth, enzyme production and adhesion of some bacteria [15, 23].

EOs, especially with known antibacterial effects, have the potential to be used in food industry as a preservative, for prevention of spoilage and to increase the shelf life of products [8, 24]. To the best of our knowledge, only a few published reports are available regarding the antimicrobial effects of the *N. cataria* EOs, especially against food-borne and resistant microorganisms. In the present study, the chemical constituents of three different phenological stages (vegetative, floral budding and full flowering) of *N. cataria* were studied and their components were compared with each other and with the previously reported data. In addition, the antimicrobial effects of these EOs were evaluated against the common causes of food-borne infections.

## 2. Materials and Methods

### 2.1. Plant Material

This study was carried out in the research field station of the Faculty of Agriculture, Shiraz University, Iran. The station is located 1810 m above the mean sea level, with the latitude of 29°36′ north and altitude of 52°32′ east. The minimum and maximum temperatures of the field in the recent ten years were −10°C and 38°C, respectively. The daily climatic data during this study were obtained from the agro-meteorological station of Irrigation Department located in a state farm about 500 m far from the experimental site. Catnip seeds (obtained from Medicinal Plants and Drugs Research Institute, Shahid Beheshti University, Iran) were sown in January 2010 in a sandy-loam textured soil with pH = 7.5, EC = 1.8 dS m^−1^, 0.97% organic matter, 0.094% N, 24 ppm P, 250 ppm K, 4.5 ppm Fe, 0.42 ppm Zn, 20 ppm Mn, and 0.94 ppm Cu. The plant samples were harvested at vegetative, floral budding, and full flowering stages. The plant species was identified and authenticated by A. R. Khosravi, a plant taxonomist, at Shiraz University, Herbarium, Shiraz, Iran. Voucher specimen (no. 24995) has been deposited in the herbarium. 

### 2.2. Essential Oil Extraction

The aerial parts of the plants were harvested at vegetative, floral budding, and full flowering stages, and then air dried. The samples (30 g, three replicates for each stage) were hydro-distillated for 3 hr using an all glass Clevenger-type apparatus, to extract EOs, according to the method recommended by the European Pharmacopoeia [25]. The extracted EO samples were dried over anhydrous sodium sulphate and stored in sealed vials at low temperature (4°C) before gas chromatography (GC) and gas chromatography/mass spectrometric (GC/MS) analysis.

### 2.3. GC and GC/MS Analysis

The analysis of EOs was carried out using a Thermoquest-Finnigan Trace GC-MS instrument equipped with a DB-5 fused silica capillary column (60 m × 0.25 mm i.d., film thickness 0.25 mm). The oven temperature was programmed to increase from 60 to 250°C at a rate of 4°C min^−1^ and finally held for 10 min; transfer line temperature was 250°C. Helium was used as the carrier gas at a flow rate of 1.1 mL min^−1^ with a split ratio equal to 1/50. The quadrupole mass spectrometer was scanned over the 35–465 amu with an ionizing voltage of 70 eV and an ionization current of 150 mA.

### 2.4. Chromatography Flame Ionisation Detector (GC/FID) Analyses

The GC/FID analysis of the oils was conducted using a Thermoquest-Finnigan instrument equipped with a DB-5 fused silica column (60 m × 0.25 mm i.d., film thickness 0.25 mm). Nitrogen was used as the carrier gas at the constant flow rate of 1.1 mL min^−1^; the split ratio was the same as that used for GC/MS. The oven temperature was raised from 60 to 250°C at a rate of 4°C min^−1^ and held for 10 min. The injector and detector (FID) temperatures were kept at 250 and 280°C, respectively. Semiquantitative data were obtained from FID area percentages without the use of correction factors.

### 2.5. Identification of Essential Oil Components

Retention indices (RI) were calculated using retention times of n-alkanes (C6–C24) that were injected after the oil at the same temperature and conditions. The compounds were identified by comparison of their RI with those reported in the literature, and their mass spectrum was compared with the Wiley Library [26].

### 2.6. Determination of Antimicrobial Activities

#### 2.6.1. Microorganisms

The antimicrobial activities of the EOs against food-borne pathogens including standard species of *S. aureus* (ATCC 29213 and ATCC 700698), *Escherichia coli* (ATCC 25922), enterohemorrhagic *E. coli* (ATCC 43894), *Shigella flexneri* (NCTC 8516), *Bacillus cereus* (ATCC 11778), *P. aeruginosa* (ATCC 27853), *Aspergillus flavus* (ATCC 64025), *A. fumigates* (ATCC 14110 and CBS 144.89), *A. clavatus* (CBS 514.65), *A. orizae* (CBS 818.72), and clinical isolates of *S. aureus*, *E. coli*, *Shigella* spp, *Salmonella* spp*, Listeria monocytogenes* and *P. aeruginosa* collected from Dr. Faghihi Hospital (Shiraz, Iran) were determined in this study. The susceptibility of all clinical isolates of bacteria and fungi against selected antibiotics was examined by microdilution and disk diffusion methods [27–29].

#### 2.6.2. **Determination of Minimum Inhibitory Concentration (MIC)**

MICs were determined using broth microdilution method recommended by the CLSI with some modifications [28, 29]. Briefly, for determination of antifungal activities against filamentous fungi, serial dilutions of the EOs (0.031 to 16.0 *μ*L/ml) were prepared in 96-well microtitre plates using RPMI-1640 media (Sigma, St. Louis, USA) buffered with MOPS (Sigma, St. Louis, USA). To determine the antibacterial activities, serial dilutions of the EOs (0.125 to 128.0 *μ*L/ml) were prepared in Muller-Hinton Broth media (Merck, Darmstadt, Germany). Test fungi or bacteria strains were suspended in the media and the cell densities were adjusted to 0.5 McFarland standards at 530 nm wavelength using a spectrophotometric method (this yields stock suspension of 1–5 × 10^6^ cells/mL for fungi and 1–1.5 × 10^8^ cells/mL for bacteria). 0.1 mL of the working inoculums was added to the microtiter plates, which were incubated in a humid atmosphere at 30°C for 24–48 h (fungi) or at 37°C for 24 h (bacteria). 200 *μ*L of the uninoculated medium was included as a sterility control (blank). In addition, growth controls (medium with inoculums but without essential oil) were also included. The growth in each well was compared with that of the growth control well. MICs were visually determined and defined as the lowest concentration of the essential oil produced no visible growth. Each experiment was performed in triplicate. In addition, media from wells with fungi showing no visible growth were further cultured on Sabouraud Dextrose Agar (Merck, Darmstadt, Germany) and from wells with bacteria showing no visible growth on Muller-Hinton agar (Merck, Darmstadt, Germany) to determine the minimum fungicidal concentration (MFC) and minimum bactericidal concentration (MBC). MBCs and MFCs were determined as the lowest concentration yielding no more than 4 colonies, which corresponds to a mortality of 99.9% of the microbes in the initial inoculums.

## 3. Results and Discussion

### 3.1. Results

The hydrodistillation of 30 g of the aerial parts of *N. cataria* at the vegetative, floral budding, and full flowering stages yielded 0.3, 0.5, and 0.9% (w/w) essential oil, respectively. The composition of EOs at different growth stages is shown in Table 1 in the order of their elution from a DB-5 column. A total of 13, 12, and 14 compounds representing 93.6, 99.6, and 99% of the total were detected at vegetative, floral budding, and full flowering stages, respectively. The nepetalactones including 4a-*α*,7-*α*,7a-*β*-nepetalactone (55–58%) and 4a-*α*,7-*β*,7a-*α*-nepetalactone (30–31.2%) were the major oil constituents of all growth stages. The highest amount of components such as *α*-pinene (4.6%), *β*-pinene (1.64%), and 4a-*α*,7-*β*,7a-*α*-nepetalactone (31.2%) were detected at full flowering stage. 

The antibacterial activities of the EOs of *N. cataria* against the common causes of food-borne infections are shown in Table 2. The EOs inhibited the growth of all Gram-positive food-borne bacteria at concentrations of 0.125–1 *μ*L/mL. Furthermore, the EOs exhibited the bactericidal activity (MBC) for all of the above-mentioned Gram-positive bacteria at concentrations ranging from 0.5 to 8 *μ*L/mL. All of the Gram-negative bacteria were susceptible to the Catnip oils at concentrations of 0.25–32 *μ*L/mL. The essence of *N. cataria* at different growth stages exhibited bacteriostatic and bactericidal activities against the *Shigella* and *Salmonella* species at concentrations of 0.25–2 *μ*L/mL and 0.5–8 *μ*L/mL, respectively. Moreover, the EOs inhibited the growth of the standard species of *Aspergillus* at concentration of 0.25–1 *μ*L/mL.

### 3.2. Discussion

The composition of the EOs might be affected by the developmental stages and geographical region of the plant [5, 7, 24]. Similar to the previous reports [15–20], we identified nepetalactone isomers as the major constituent of the Catnip EOs in all stages of growth, which reached its maximum level at the floral budding stage. However, some studies have detected no nepetalactones in their examined EOs and reported 1,8-cineol [30] and alpha-citral [31] as the most abundant compounds of the catnip oil. The pinene (*α* and *β*) was detected as the third main component of the EO in the present work, increasing gradually following maturation of the plant. 

Food-borne pathogens are considered as one of the leading causes of gastrointestinal diseasesand mortality in less developed and developing countries [32]. In the present study, the main causes of food-borne infections including *S. aureus*, *B. cereus, L. monocytogenes*, *E. coli*, *Shigella* spp. and *Salmonella* spp. were inhibited by the EOs at concentration of 0.125–2 *μ*L/mL. The results can be best compared to those of the study reported by Zenasni et al. on the same strains [17]. Many of the filamentous fungi, in particular *Aspergillus* species, are known for causing food spoilage and mycotoxin production. Similar to the study of Adiguzel et al., the growth of the examined *Aspergillus* spp. was inhibited at concentrations up to 0.5 *μ*L/mL [15]. 

In the present study, the MICs of the EOs against Gram-positive bacteria were almost lower than those of Gram-negative bacteria. No significant difference was found in the MICs of the EOs between antibiotics-susceptible and antibiotics-resistant strains, suggesting that the mechanism of the action of the EO might be different from that of the tested antibiotics. As expected from the results of GC/MS analysis, no significant differences in MICs were found between the EOs distilled from different growth stages. 

As nepetalactone isomers were detected as the major compounds of catnip EO, the good antimicrobial properties of the EO found in this study and the other reports might be the characteristic of nepetalactones [15, 17, 19]. However, further studies are still needed to clarify the antimicrobial properties of the isomers of nepetalactone molecule. 

## 4. Conclusion 

Nowadays there is a great demand to reduce the use of chemical preservatives in products, EOs of *N. cataria* with active antimicrobial properties might be a candidate as a natural source for the maintenance or extension of the shelf life of products. In addition, delectable taste and odor of the EOs is an additional benefit to its antimicrobial activities. Moreover, the EOs of *N. cataria* might be considered for developing antimicrobial agents and disinfectants. However, further studies especially in animal models are still required to determine the in vivo antimicrobial activity of the EO and its ingredients. 

## Figures and Tables

**Table 1:** EO composition (%) of catnip (*N. cataria*) at three stages of harvest.

Component	RI^a^	Vegetative (%)	Floral budding (%)	Full flowering (%)
*α*-Pinene	936	2.7	3.1	4.6
Sabinene	957	0.0	0.0	0.15
*β*-Pinene	978	0.8	1.13	1.64
1-Cyclohexen-1-yl-methyl ketone	980	0.5	0.6	0.7
Triplal	1023	0.1	0.4	0.4
Thymol	1294	0.4	0.4	0.57
4a-*α*,7-*α*,7a-*β*-Nepetalactone	1332	55	58	55.03
4a-*α*,7-*β*,7a-*α*-Nepetalactone	1342	30.06	31.1	31.2
trans Caryophyllene	1430	1.1	2.7	2.1
*α*-Humulene	1446	0.82	0.92	0.87
11-Dodecenol	1500	0.84	0.69	1.1
Spathulenol	1580	0.6	0.4	0.3
Caryophyllene oxide	1569	0.5	0.2	0.12
6,10-dimethyl-2-undecane	1907	0.2	0.0	0.25

^a^Retention index.

**Table 2:** Antimicrobial activity (MIC and MBC) of essential oils distilled from *N. cataria*'*s* stages against food-borne pathogens.

		Stage 1		Stage 2		Stage 3	
Bacteria (number of strains)		MIC^a^ (*μ*L/mL)	MMC^b^ (*μ*L/mL)	MIC^a^ (*μ*L/mL)	MMC^b^ (*μ*L/mL)	MIC^a^ (*μ*L/mL)	MIC^b^ (*μ*L/mL)
		GM^c^ (range)	GM^c^ (range)	GM^c^ (range)	GM^c^ (range)	GM^c^ (range)	GM^c^ (range)
Bacteria	Methicillin resistant *S. aureus* (6)	0.22 (0.125–0.5)	1.12 (1-2)	0.17 (0.125–0.5)	1.41 (1-2)	0.28 (0.25–0.5)	1.41 (1-2)
	Methicillin sensitive *S. aureus* (6)	0.19 (0.125–0.5)	0.89 (0.5–2)	0.15 (0.125–0.25)	1.26 (1-2)	0.22 (0.125–0.5)	1.41 (1–4)
	*B. cereus* ATCC 11778	1	2	1	1	0.5	1
	*L*. *monocytogenes *	1	4	1	8	1	8
	Third generation cephalosporins resistance *E. coli* (5)	3.48 (2–4)	6.96 (4–8)	3.48 (2–4)	6.96 (4–8)	3.03 (2–4)	5.65 (2–8)
	Third generation cephalosporins sensitive *E. coli* (6)	4 (2–8)	8.98 (4–16)	3.56 (2–8)	8.98 (4–16)	3.17 (2–4)	6.35 (2–8)
	Enterohemorrhagic *E. coli* ATCC 43894	1	2	1	2	1	1
	*S. flexneri* NCTC 8516	0.5	2	0.5	2	0.5	2
	*S*. *dysentery* (4)	0.5 (0.25–2)	2 (1–4)	0.5 (0.25–2)	1.68 (1-2)	0.42 (0.25–2)	1 (0.5–2)
	*S*. *sonnei *	2	8	1	2	1	2
	*S. enterica* serovarparatyphi A	2	4	2	4	1	2
	*S. enterica* serovarparatyphi B	2	4	2	4	1	2

Fungi	*A. flavus*ATCC 64025	0.5	1	0.5	1	0.5	1
	*A. fumigatus* ATCC 14110	0.5	1	0.5	1	0.5	1
	*A. fumigatus* CBS 144.89	0.5	1	0.5	1	0.5	1
	*A. clavatus* CBS 514.65	0.5	1	0.5	1	0.25	1
	*A. orizae* CBS 818.72	0.5	1	0.5	1	0.5	1

^a^ Minimum inhibitory concentration, ^b^Minimum microbicidal concentration, ^c^Geometric mean.
